# Enhanced Recovery After Surgery (ERAS) Protocols in Sarcoma and Metastatic Bone Disease: A Scoping Review

**DOI:** 10.3390/cancers18213424

**Published:** 2026-10-22

**Authors:** Leah Ferrie, Alex Gamage, Peter Lewkonia, Michael M. H. Yang, Khara M. Sauro, Krista Goulding, Shannon K. T. Puloski, Michael J. Monument, Joseph K. Kendal

**Affiliations:** 1 Department of Surgery, Section of Orthopaedic Surgery, University of Calgary, Calgary, AB T2N 1N4, Canada; 2 Department of Surgery, Cumming School of Medicine, Calgary, AB T2N 4Z6, Canada; 3 Department of Clinical Neurosciences, Cumming School of Medicine, Calgary, AB T2N 4Z6, Canada; 4 Department of Community Health Sciences, Cumming School of Medicine, Calgary, AB T2N 4Z6, Canada; 5 Department of Oncology, Cumming School of Medicine, Calgary, AB T2N 4Z6, Canada; 6 Department of Orthopaedic Surgery, Mayo Clinic Arizona, Scottsdale, AZ 85259, USA

**Keywords:** enhanced recovery, ERAS, fast-track, sarcoma, metastatic bone disease

## Abstract

**Simple Summary:**

Enhanced Recovery After Surgery (ERAS) pathways have been widely implemented across surgical specialties, with strong evidence demonstrating their impact on improving patient outcomes. This study aims to synthesize the current state of literature and identify gaps in the application of ERAS in orthopaedic oncology. Using the Arksey and O’Malley framework for scoping reviews, a comprehensive literature search was performed, and nine studies meeting eligibility criteria were identified. Data from selected studies were extracted, analyzed, and summarized using descriptive analytics. ERAS pathways in orthopaedic oncology were found to be in an early stage of development, primarily concentrated in spine oncology, with few studies in non-spine sarcoma and appendicular metastatic bone disease. There was also notable variability in ERAS elements included and outcomes reported across studies. These results present the opportunity for further work in developing specific orthopaedic oncology ERAS pathways that incorporate core principles, standardized perioperative outcomes, and measures of pathway adherence to improve outcomes in this complex patient population.

**Abstract:**

Literature evaluating the effectiveness of Enhanced Recovery After Surgery (ERAS) pathways in orthopaedic oncology, particularly in bone and soft tissue sarcomas, is varied and has not been synthesized to understand the current state of evidence. However, substantial efforts have been made to evaluate ERAS in other orthopaedics procedures, particularly in hip and knee arthroplasty. This study aims to summarize the breadth and depth of literature on ERAS pathways in orthopaedic oncology to identify critical gaps and guide future research directions given the potential benefits in this complex population. The Arksey and O’Malley framework in accordance with the Preferred Reporting Items for Systematic Reviews and Meta-Analyses extension for Scoping Reviews (PRISMA-ScR) was used to generate a search strategy, define eligibility, and select studies from a comprehensive literature search. Of the nine included studies, data were extracted and analyzed using descriptive analytics. The available literature was heterogeneous; it predominantly comprised retrospective studies, concentrated on spine and spinopelvic oncology, ERAS components included, and reported outcomes (although length of stay was assessed in all included studies). Comparatively little evidence was identified in non-spine sarcoma, and no included study specifically evaluated appendicular metastatic bone disease. These findings highlight the need for prospective disease- and surgery-specific ERAS implementation studies using standardized pathway definitions, adherence measures, and perioperative outcomes.

## 1. Introduction

Enhanced Recovery After Surgery (ERAS) protocols are evidence-based perioperative care pathways designed to standardize care, accelerate functional recovery, and improve postoperative outcomes. ERAS protocols span preoperative, intraoperative, and postoperative care, and they have been widely implemented across many surgical disciplines including colorectal surgery, urology, and elective orthopaedic surgery [1]. In hip and knee arthroplasty, ERAS protocols have been associated with reduced length of stay (LOS), reduced intraoperative blood loss, lower 30-day postoperative mortality, and better early functional recovery [2,3,4].

Despite these advances, the role of ERAS in orthopaedic oncology remains poorly understood [5]. Patients undergoing surgery for soft tissue sarcoma or metastatic bone disease (MBD) represent a heterogeneous population with unique challenges including frailty, malnutrition, anaemia, and ongoing perioperative radiation and systemic therapy. Furthermore, these patients often undergo complex resections and reconstructions, have relatively high rates of wound complications and venous thromboembolism, and struggle with impaired mobility and struggle with impaired mobility and variable needs, creating challenges in discharge planning [6,7,8]. Oncologic surgeries are often structured around patients’ complex care needs, which can make adhering to ERAS protocols challenging while simultaneously delivering patient-centred care. These factors make standardized perioperative pathways more difficult to design and implement.

ERAS has been consistently shown to reduce hospital LOS, which is a salient outcome among patients undergoing orthopaedic oncology surgeries [9]. LOS is a particularly relevant outcome in orthopaedic oncology. Prolonged inpatient recovery may not only reflect patient comorbidities and surgical complexity, but is also affected by complications, impaired mobility, wound concerns, and discharge barriers, while also potentially delaying return to multidisciplinary oncologic care. In a secondary analysis of the Prophylactic Antibiotic Regimens in Tumor Surgery (PARITY) trial, hospital LOS was the only independent predictor of surgical site infection following lower extremity endoprosthetic reconstruction [7]. These findings highlight further that LOS is not simply a measure of hospital efficiency in orthopaedic oncology, but an important marker of perioperative recovery and support efforts to develop streamlined, multidisciplinary perioperative pathways in this population.

Although the number of studies on ERAS has increased in recent years, there remains limited evidence in orthopaedic oncology, which requires further assessment [9]. Furthermore, existing studies often focus on specific tumour types, anatomic site (which in turn have varied operative complexity), pathway components, and different outcomes. Because of the heterogeneity among studies, the benefits of ERAS protocols remain unclear. Moreover, it is uncertain whether the current literature adequately captures recovery outcomes that are most relevant to patients with sarcoma and MBD, including pain, opioid use, mobilization, discharge disposition, complications, readmission, return to systemic therapy, quality of life, and functional recovery.

The objective of this scoping review was to summarize the current literature on ERAS pathways in orthopaedic oncology, with a focus on patients undergoing surgery for sarcoma and MBD. Specifically, this review aimed to characterize the populations studied, describe the ERAS elements implemented, summarize postoperative outcomes, and highlight any gaps in the existing evidence to guide future research.

## 2. Methods

This scoping review was conducted using the methodological framework described by Arksey and O’Malley; it has been reported in accordance with the Preferred Reporting Items for Systematic Reviews and Meta-Analyses extension for Scoping Reviews (PRISMA-ScR) and has not been registered [10].

### 2.1. Search Strategy

A comprehensive literature search was performed using MEDLINE and EMBASE databases from database inception to August 2024, with the assistance of a librarian. Search terms used included combinations of keywords related to orthopaedic oncology populations and perioperative recovery pathways including “bone neoplasms”, “neoplasm metastasis”, “sarcoma”, “multiple myeloma”, “enhanced recovery after surgery”, “early recovery pathway”, “protocol”, “framework”, or a combination of these terms. The full search strategy is included in Appendix A.

### 2.2. Eligibility Criteria

Eligible studies were included in the scoping review if the following criteria were met: (1) involved human patients undergoing surgery for soft tissue sarcoma, bone sarcoma, metastatic bone disease, or multiple myeloma; (2) evaluated an ERAS protocol, fast-track pathway, postoperative recovery protocol, clinical practice guideline (CPG), or enhanced perioperative care pathway as explicitly identified and described by the study authors (formal ERAS society certification or adherence to a prespecified number of ERAS components was not required for inclusion); and (3) reported at least one postoperative outcome of interest, such as LOS, adverse events, complications, pain, analgesic use, mobilization, readmission, discharge disposition, or patient-reported outcomes. Studies involving only benign bone or soft tissue tumours were excluded. Conference abstracts, editorials, letters to the editors, narrative reviews, and systematic reviews were also excluded. Only studies in English were included.

### 2.3. Study Selection

Covidence was used to screen search results for duplicate removal, title and abstract screening, and full-text review. Two reviewers independently screened titles and abstracts for eligibility (L.F. and A.G.). The full-text article of studies identified as potentially eligible in title and abstract screening were then reviewed independently by the same reviewers to determine final eligibility. Disagreements were resolved through discussion, with a third reviewer to settle any unresolved conflicts (J.K.). Cohen’s kappa for full-text review was 0.92.

### 2.4. Data Collection Process

This review was focused on ERAS pathways and perioperative and early postoperative recovery outcomes associated with surgery. Longer-term patient-reported, functional, and oncologic outcomes were extracted when reported, but were not the primary focus of the search strategy. Data charting was conducted using a standardized data extraction form. Extracted variables included author, year, country of study, journal name, study design, patient population, sample size, mean age, sex, tumour type, anatomic site, setting, ERAS pathway components, comparator group when applicable, outcomes measured, and key findings. ERAS elements were categorized as preoperative, intraoperative, and postoperative components.

### 2.5. Risk of Bias in Individual Studies

Given the scoping nature of this review and the expected heterogeneity of study designs, interventions, and outcomes, formal risk-of-bias assessment and meta-analysis were not performed. Results were synthesized descriptively based on tumour type, ERAS pathway components, outcomes, and key findings.

## 3. Results

### 3.1. Study Selection

**Figure 1:**
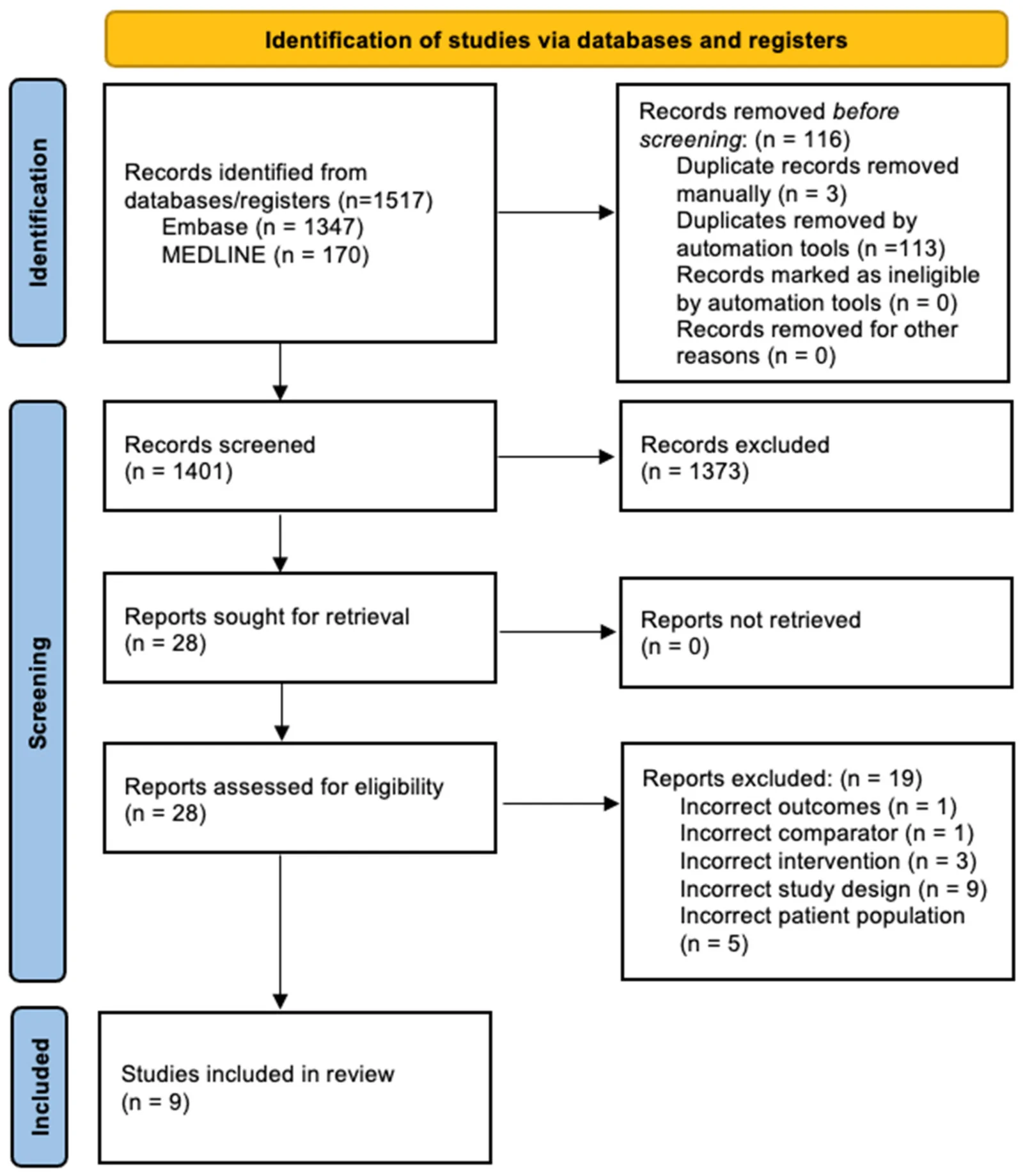
PRISMA flow diagram illustrating study selection process.

The search identified 1517 studies from MEDLINE and Embase. After duplicates were removed and title and abstract were screened, 28 full-text articles were assessed for eligibility. Nine studies met the eligibility criteria and were included in the scoping review (Figure 1, Table 1).

The included studies were published between 2015 and 2024 (median publication date = 2022, IQR = 2021–2023) and were conducted in France (*n* = 1), the United States (*n* = 3), China (*n* = 4), and Korea (*n* = 1) (Table 1). Study designs included matched case-control studies (*n* = 2), retrospective cohort studies (*n* = 5), retrospective case series (*n* = 1), and one prospective cohort study (*n* = 1). Most studies were single-centre investigations (*n* = 8), with one multicentre analysis of patients with metastatic epidural spinal compression (*n* = 1). Study populations were heterogeneous with respect to tumour diagnosis, anatomic site, operative complexity, and comparator groups. Several studies included mixed populations, including benign- and malignant-diagnosis primary spinal tumours and degenerative disease (*n* = 2) [5,11], or bone tumour surgery without clear differentiation between primary and metastatic disease (*n* = 1) [12]).

Among the nine included studies, eight evaluated a defined ERAS pathway [1,5,11,12,13,14,15,16], while one study evaluated a multidisciplinary perioperative protocol without a formal ERAS pathway [17]. The included studies represent 3911 patients in total. Overall, 2954 patients (76%) had a primary sarcoma, while 957 (24%) had MBD. As seen in Table 1, two studies focused primarily on soft tissue sarcoma [14,16], four studies examined spinopelvic malignancies [5,11,12,17], four included MBD of the spine [1,13,15,17], and one study also included patients with appendicular sarcomas [12]. One study included patients with benign spine tumours in their sample population without differentiating results [5].

**Figure 2:**
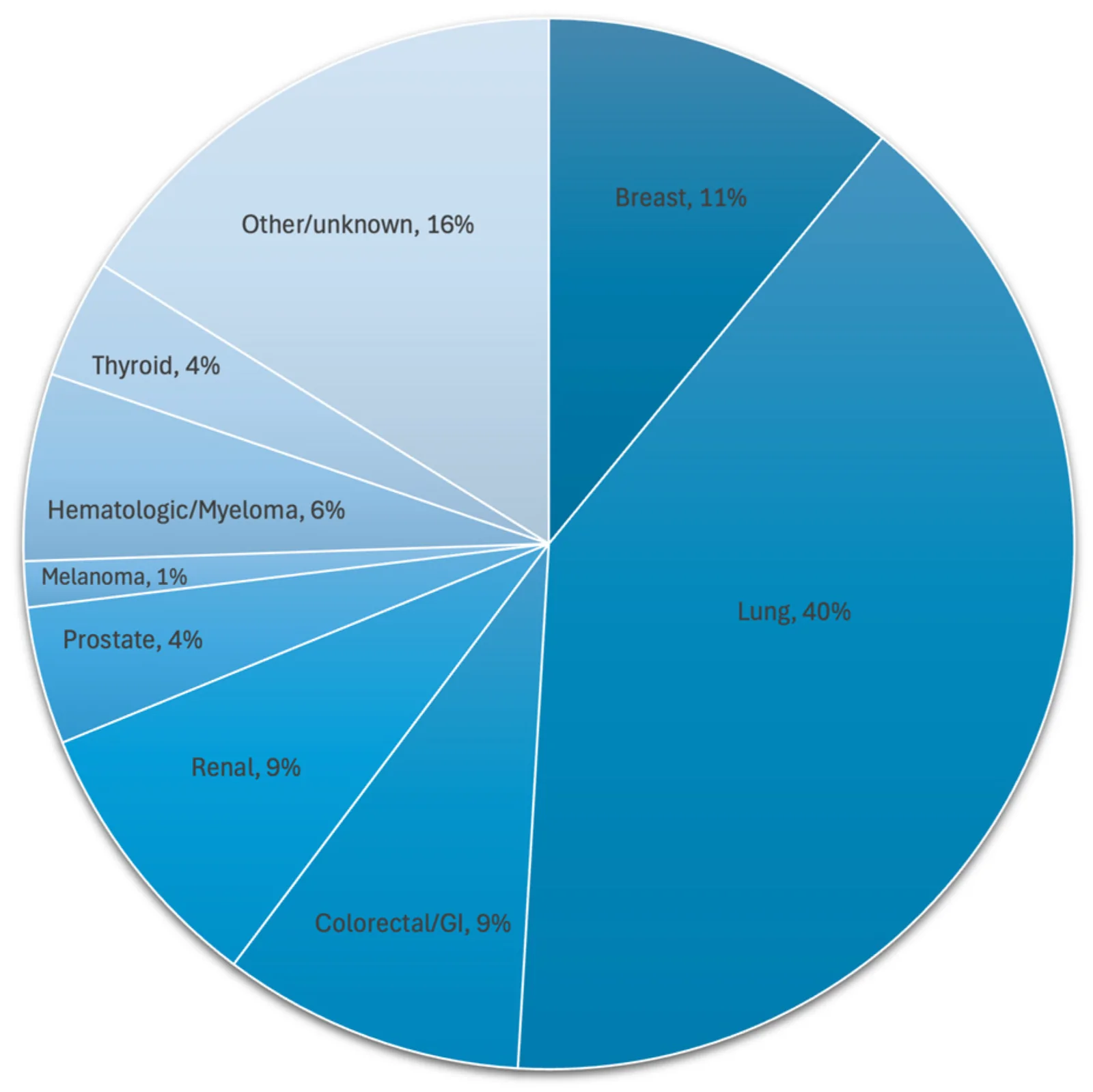
Breakdown of metastatic bone disease (MBD) primary cancer origins.

Within the MBD group (*n* = 957), the primary cancer origins were lung (*n* = 340, 36%), breast (*n* = 94, 10%), sarcoma (*n* = 83, 9%), colorectal/gastrointestinal (*n* = 79, 8%), renal (*n* = 73, 8%), hematologic/myeloma (*n* = 49, 5%), prostate (*n* = 36, 4%), thyroid (*n* = 31, 3%), melanoma (*n* = 12, 1%), and unknown/other (*n* = 161, 17%) (Figure 2).

**Table 1:** Included studies’ demographics and relevant study information.

Author	Michot et al. [16]	Lyu et al. [14]	Zhao et al. [12]	Nguyen et al. [17]	Chakravarthy et al. [1]	Wang et al. [5]	Lei et al. [13]	Yuh et al. [11]	Ma et al. [15]
Year	2015	2020	2021	2021	2022	2023	2023	2024	2024
Title	The introduction of early patient rehabilitation in surgery of soft tissue sarcoma and its impact on postoperative outcome	Enhanced recovery after surgery pathway in patients with soft tissue sarcoma	Effectiveness of enhanced recovery after surgery in the perioperative management of patients with bone surgery in China	A multidisciplinary approach to complex oncological spine coverage in high-risk patients	Patient outcomes following implementation of an enhanced recovery after surgery pathway for patients with metastatic spine tumours	Enhanced recovery after surgery (ERAS) in sacral tumour surgery and comprehensive description of a multidisciplinary program: a prospective study in a specialized hospital in China	Treatment of patients with metastatic epidural spinal cord compression using an enhanced recovery after surgery program	The iterative implementation of a comprehensive enhanced recovery after surgery protocol in all spinal surgery in Korea: a comparative analysis of clinical outcomes and medical costs between primary spinal tumours and degenerative spinal diseases	Promoting postoperative recovery in patients with metastatic epidural spinal cord compression based on the concept of ERAS: a multicentre analysis of 304 patients
Country of study	France	United States	China	United States	United States	China	China	Korea	China
Journal	EJSO	BJS	World Journal of Clinical Cases	JNS Spine	Cancer	International Orthopaedics	Frontiers in Cell and Developmental Biology	JNS Spine	The Spine Journal
Study type	Retrospective matched case control study	Retrospective matched case control study	Retrospective cohort study	Retrospective consecutive case series	Retrospective cohort study	Prospective study	Retrospective cohort study	Retrospective cohort study	Observational cohort study
Population	Adult patients (≥16 years) with primary non-metastatic STS located in the trunk wall or the limbs that were operated at Institut Bergonie between 1989 and 2012	All patients who had surgery for soft tissue sarcoma (STS) by one of three surgical oncologists from July 2015 to March 2018, with intention to treat according to this ERAS protocol	Patients undergoing bone tumour surgery between May 2019 and April 2021 at the Second Affiliated Hospital of Xi’an Jiaotong University	Patients who underwent oncological spine surgery via a multidisciplinary approach between 2015 and 2019	Patients who underwent surgery for metastatic spine disease between January 2018 and May 2020	Patients with sacral tumour (benign and malignant) between March 2021 and September 2021 at a single centre	Patients with metastatic epidural spinal cord compression (MESCC) treated with surgery at orthopaedic department of the Fifth Medical Center of PLA General Hospital (Beijing) between December 2016 and December 2022	Spine surgical procedures for adult patients with a diagnosis of primary spinal tumour (PST) or degenerative spinal disease (DSD) at the Seoul National University Hospital Spine Center performed between January 2003 and December 2021	Patients with metastatic epidural spinal cord compression (MESCC) who underwent surgery between January 2016 and January 2023 at two large tertiary hospitals
Tumour Type	Soft tissue sarcoma	Soft tissue sarcoma	Spinopelvic and appendicular sarcoma *	Spinopelvic sarcoma and MBD Spine	MBD Spine	Spinopelvic sarcoma	MBD Spine	Spinopelvic sarcoma	MBD Spine
Sample size	734	471	107	102 (primary sarcoma: 23; MBD: 79)	390	125	184	PST total: 1494 Non-ERAS (PST): 318 ERAS (PST): 1176	304
Mean age	Non-ERAS: 55 ERAS: 58	Non-ERAS: 58.5 ERAS: 58.6	Non-ERAS: 46.54 ERAS: 48.59	59.61	Mean (SD): 63.3 (11.7)	Non-ERAS: 49.6 ERAS: 45.9	Non-ERAS: 59.94 +/− 8.62 ERAS: 58.72 +/− 7.69	Not available	Non-ERAS: 61 ERAS: 59
Sex	Male: 385 Female: 349	Male: 203 Female: 268	Male: 72 Female: 35	Male: 52 Female: 50	Male: 211 Female: 179	Male: 66 Female: 59	Male: 101 Female: 83	Not available	Male: 161 Female: 143
Primary Cancer Site	Shoulder girdle: 48 Upper limb: 82 Trunk wall: 122 Pelvic girdle: 72 Lower limb: 410	Stomach: 46 Abdomen (metastatic GIST): 45 Abdomen/pelvis: 94 Retroperitoneum: 72 Lower extremity (above knee): 78 Lower extremity (below knee): 22 Upper extremity: 10 Trunk/flank: 50 Breast: 41 Head and neck: 13	Spine: 31 Pelvis: 13 Upper limb joint: 12 Lower limb joint: 51	Not specified	Breast: 33 Lung: 84 Colorectal: 37 Renal: 48 Prostate: 29 Melanoma: 12 Hematologic: 36 Unknown/other: 83 Sarcoma: 28	Sacrum	Lung: 96 Breast: 28 Prostate: 7 Digestive tract: 19 Other: 34	PST group: Intramedullary (IM): 265 Intradural extramedullary (IDEM): 890 Extradural (ED): 164 Bone: 86 Other: 89	Lung: 160 Breast: 32 Renal cell carcinoma: 25 Gastrointestinal cancer: 23 Thyroid carcinoma: 31 Myeloma: 13 Other: 20
ERAS Elements	Preadmission education Nutritional management Antithrombotic prophylaxis Anesthetic administration Postoperative analgesia Early mobilization Criteria for discharge Follow-up planning	Preadmission education Nutritional management Preemptive analgesic Intraoperative fluid management Analgesia management Management of postoperative fluids Management of postoperative nausea and vomiting Plan for opioid minimization Early mobilization Postoperative diet and bowel management	Preadmission education Nutritional management Antithrombotic prophylaxis Preemptive analgesic Blood management Anesthetic administration Intraoperative fluid management Bleeding control Analgesia management Management of postoperative fluids Postoperative analgesia Management of postoperative nausea and vomiting Urinary catheter discontinuation Early mobilization Postoperative diet and bowel management Follow-up planning	Nutritional management	Preadmission education Preemptive analgesia Intraoperative fluid management Analgesia management Postoperative analgesia Urinary catheter discontinuation Early mobilization Postoperative diet and bowel management	Preadmission education Nutritional management Blood management Bleeding control Analgesia management Postoperative analgesia Management of postoperative nausea and vomiting Drain and line management Early mobilization Postoperative diet and bowel management Follow-up planning	Preadmission education Nutritional management Antithrombotic prophylaxis Preemptive analgesia Blood management Anesthetic administration Intraoperative fluid management Bleeding control Analgesia management Management of postoperative fluids Postoperative analgesia Management of postoperative nausea and vomiting Plan for opioid minimization Drain and line management Urinary catheter discontinuation Early mobilization Postoperative diet and bowel management	Preadmission education Nutritional management Anesthetic administration Intraoperative fluid management Analgesia management Postoperative analgesia Management of postoperative nausea and vomiting Plan for opioid minimization Early mobilization Postoperative diet and bowel management Follow-up planning	Preadmission education Nutritional management Antithrombotic prophylaxis Preemptive analgesic Antiemetic prophylaxis Blood management Anesthetic administration Intraoperative fluid management Bleeding control Analgesia management Management of postoperative fluids Postoperative analgesia Management of postoperative nausea and vomiting Plan for opioid minimization Drain and line management Urinary catheter discontinuation Early mobilization Postoperative diet and bowel management
Outcomes measured	LOS Bleeding and intraoperative blood loss Wound infection Patient satisfaction Drainage Wound dehiscence Seroma formation Re-admission Overall survival (OS)	LOS Bleeding and intraoperative blood loss Wound infection Return to regular diet Wound dehiscence Seroma formation Re-admission Intraoperative fluid intake Blood transfusions Ileus and/or obstruction	LOS Wound infection Time to ambulation Patient satisfaction Drainage Postoperative pain Blood transfusions Mortality PONV	LOS Wound infection Drainage Wound dehiscence Seroma formation Mortality	LOS Bleeding and intraoperative blood loss Time to ambulation Return to regular diet Urinary catheterization Intraoperative fluid intake Postoperative pain medication use	LOS Bleeding and intraoperative blood loss Wound infection Time to ambulation Drainage Re-admission Ileus and/or obstruction	LOS Bleeding and intraoperative blood loss Time to ambulation Patient satisfaction Return to regular diet Urinary catheterization	LOS Cost	LOS Bleeding and intraoperative blood loss Wound infection Time to ambulation Patient satisfaction Return to regular diet Wound dehiscence Postoperative pain Urinary catheterization
Notes			* Unclear whether patient population with bone tumours have primary sarcoma or metastatic cancer to bone. This is not specified, so our assumption is primary sarcoma for the purpose of this analysis.	No comparator group—not an ERAS vs. non-ERAS study. Only looks at outcomes from implementation of their protocol.		Population includes benign tumours in sample population without differentiating in Section 3		Population included is not exclusive to cancer—includes degenerative spinal disease (DSD) which for the purposes of the results reported was not included	

### 3.2. ERAS Components

**Figure 3:**
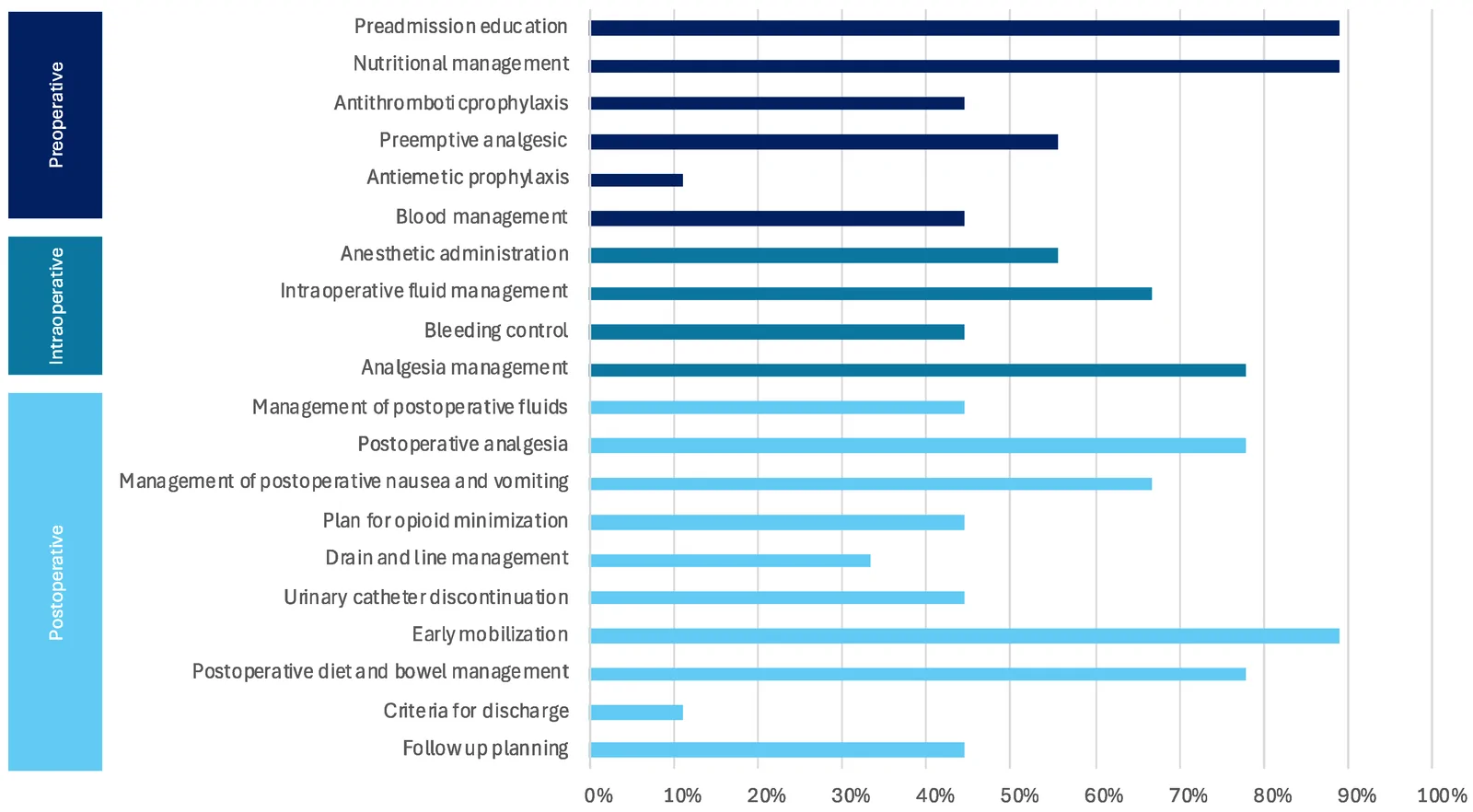
ERAS components from included studies.

There was substantial variability in ERAS elements reported across included studies, as shown in Figure 3. All studies except for that of Nguyen et al. [17] used a defined ERAS or enhanced recovery pathway, although the specific components and level of detail varied considerably (Appendix A.2 Table A1). ERAS categories included were adapted from commonly reported ERAS elements throughout the available literature [9,18,19]. The ERAS elements most commonly included were preadmission or preoperative patient education (*n* = 8, 89%), preoperative nutritional assessment or optimization (*n* = 8, 89%), and postoperative early mobilization (*n* = 8, 89%). Intraoperative analgesia management, postoperative analgesia, and postoperative diet and bowel management were each reported in 78% of the studies (*n* = 7). Less frequently reported elements included antiemetic prophylaxis and explicit discharge criteria, each reported in 11% of studies (*n* = 1).

### 3.3. Outcomes Measured

**Figure 4:**
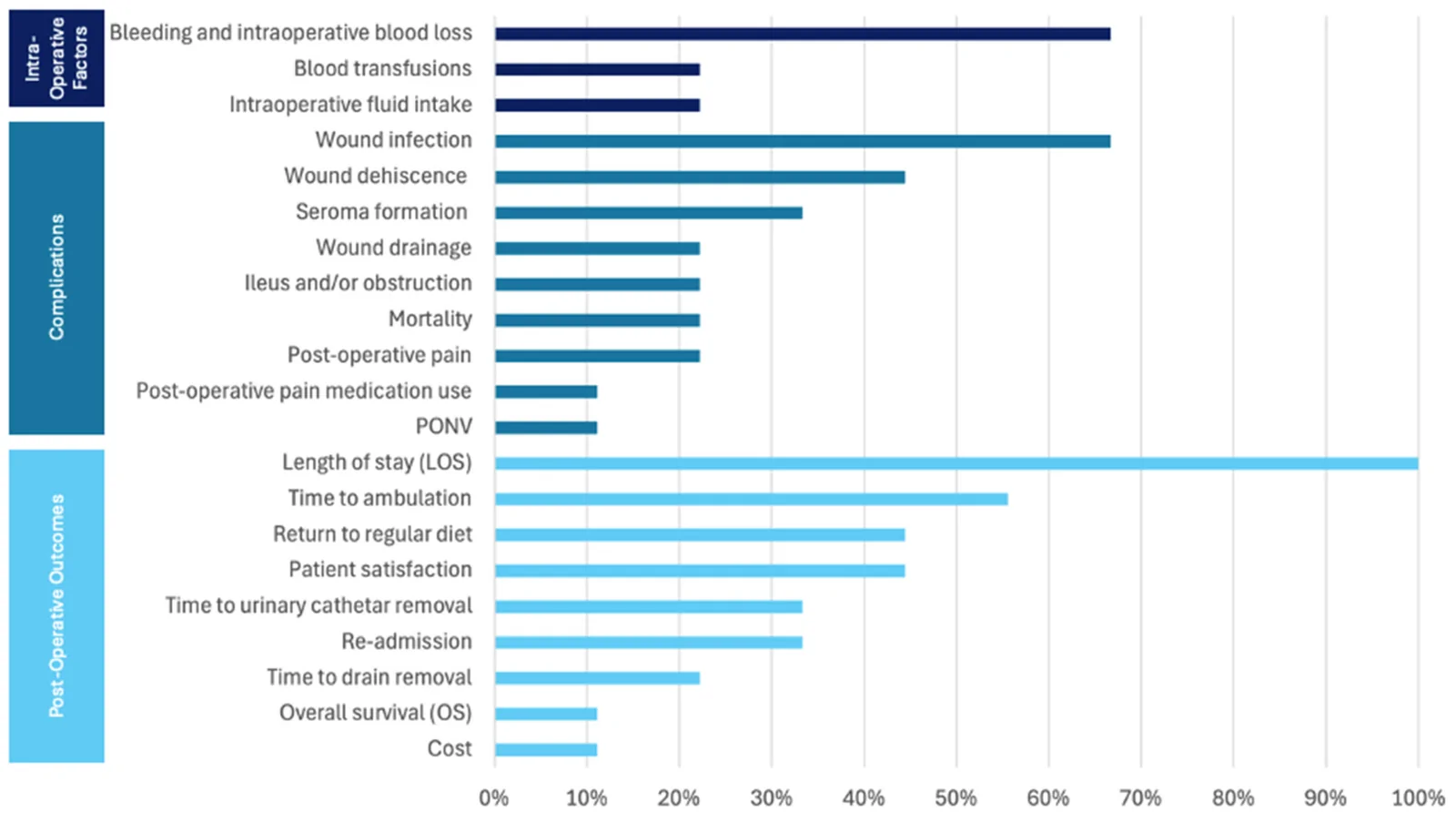
Frequency of outcomes measured across included studies.

Reported outcomes varied substantially across studies (Figure 4). LOS was the only outcome reported by all nine studies (*n* = 9, 100%). Other commonly reported outcomes included bleeding or perioperative blood loss (*n* = 6, 67%), wound infection (*n* = 6, 67%), and time to ambulation (*n* = 5, 56%). Less frequently reported outcomes included patient satisfaction (*n* = 4, 44%), return to regular diet (*n* = 4, 44%), urinary catheter duration (*n* = 3, 33%), drain duration (*n* = 2, 22%), wound drainage (*n* = 2, 22%), wound dehiscence (*n* = 4, 44%), readmission (*n* = 3, 33%), postoperative pain (*n* = 2, 22%), opioid or analgesic use (*n* = 1, 11%), cost (*n* = 1, 11%), overall survival (*n* = 1, 11%), and mortality (*n* = 2, 22%).

Notably, patient-centred and oncologic recovery outcomes were inconsistently reported. Fewer than half of included studies assessed postoperative pain (*n* = 2, 22%), analgesic use (*n* = 1, 11%), readmission (*n* = 3, 33%), discharge disposition (*n* = 0, 0%), quality of life (*n* = 0, 0%), or overall survival (*n* = 1, 11%). Consistent with the perioperative focus of ERAS, most reported outcomes reflected early surgical recovery. Longer-term patient-reported, functional, and oncologic outcomes were less frequently evaluated. This heterogeneity limited direct comparison across studies, and summarizing the data with descriptive statistics was not possible.

## 4. Discussion

This scoping review identified a limited but growing body of literature evaluating ERAS pathways in orthopaedic oncology. Overall, the literature was dominated by retrospective studies, spine or spinopelvic oncology populations, and hospital-based recovery outcomes, with limited evidence specific to non-spine sarcoma, appendicular MBD, validated patient-reported outcomes, or longitudinal functional recovery.

There was considerable variability amongst ERAS elements reported across the nine studies. The studies contained commonly included elements of preoperative education, preoperative nutrition, perioperative multimodal analgesia, and early postoperative mobilization, but substantial variability remained in other pathway components and standardized recovery milestones, such as antiemetic prophylaxis and explicit discharge criteria. Given the relevance of nutritional status in major sarcoma surgery, perioperative nutrition was shown to be an important element, as it was reported in 89% of included studies. Samà et al. recently evaluated the prognostic impact of perioperative nutritional status in retroperitoneal sarcoma patients, illustrating the importance of perioperative nutrition optimization as a modifiable factor in reducing postoperative complications and improving outcomes [20].

Across nine studies, ERAS pathways most commonly reported LOS, time to mobilization, pain control, blood loss or transfusion requirements, return to diet, and complications. However, the available evidence remains heterogeneous, largely retrospective, and concentrated in spine and spinopelvic populations. Comparatively few studies have evaluated ERAS pathways in soft tissue or bone sarcoma, and no included study specifically assessed use of ERAS in appendicular-only MBD. These findings highlight an important gap in the evidence around the adoption and effectiveness of ERAS in orthopaedic oncology. The principal research gaps identified by this review and corresponding priorities for future investigation are summarized in Table 2.

The concentration of enhanced recovery pathways in spine oncology is notable but not unexpected. Spine surgery has increasingly adopted multidisciplinary perioperative pathways due to the high morbidity associated with complex reconstruction, neurologic compromise, postoperative pain, blood loss, delayed mobilization, and prolonged hospitalization [21]. These challenges are amplified in patients with metastatic spine disease or those with epidural cord compression for whom perioperative care must also account for frailty, corticosteroid use, radiotherapy, systemic therapy, neurologic status, and limited projected survival [22]. ERAS protocols in these populations provide a framework for standardizing multidisciplinary perioperative care, with emerging evidence suggesting improved perioperative outcomes. A recent systematic review and meta-analysis identified that the use of ERAS in patients with spinal metastases resulted in a shorter LOS, decreased operative blood loss, earlier time to ambulation, and earlier time to radiation and/or systemic therapy [23]. More recent work has emphasized this with a 2026 consensus review on ERAS for spinal metastasis identifying 19 ERAS items across the care continuum, using available evidence to create recommendations for care [24].

In contrast, the evidence and recommendations for pelvic and appendicular MBD and sarcoma ERAS pathways are less mature. This gap is clinically relevant as these populations face many of the same challenges that originally motivated ERAS development. However, orthopaedic oncology populations are highly heterogeneous, which may make standardized pathways more difficult than in comparative orthopaedic populations such as those undergoing hip and knee arthroplasty. Patients undergoing sarcoma or MBD surgery vary substantially by tumour biology, prognosis, anatomic site, use of adjunctive oncologic therapies, reconstruction strategy, soft tissue reconstruction, postoperative activity restrictions, and goals of care [25]. Furthermore, patients with MBD may present urgently with pathologic fracture or uncontrolled pain, limiting opportunities for preoperative education, nutritional optimization, and other preoperative ERAS elements, while chemotherapy or radiotherapy may similarly influence the feasibility and timing of individual pathway components. A single uniform ERAS pathway may therefore be less appropriate than a framework that incorporates core ERAS principles while allowing adaptation to diagnosis, surgical intervention, expected survival, and functional goals. The principal gap in the current literature may therefore be less whether ERAS can improve perioperative recovery in orthopaedic oncology, for which early evidence is encouraging [1,5,12,13,15,16], and more how ERAS should be defined, adapted, implemented, and evaluated across a highly heterogeneous oncology population.

Length of stay was the only outcome reported across all studies included in our scoping review. This is consistent with the broader ERAS literature, where LOS is commonly used as a marker of recovery, hospital efficiency, and pathway effectiveness [9]. In MBD surgery, LOS may reflect patient frailty, elective or emergent presentation, pain, medical complexity, discharge barriers, and limited physiologic reserve; recent evidence demonstrating substantially longer LOS following emergent compared with elective MBD surgery further illustrates the influence of care pathways and presentation on this outcome [8]. In contrast, for many elective sarcoma resections, particularly in the extremity, reduced LOS may represent a meaningful marker of successful perioperative optimization, early mobilization, pain control, and discharge planning. This distinction is important because LOS should not be compared across fundamentally different procedures, such as an internal hemipelvectomy and limited extremity soft tissue sarcoma resections, without stratification by operative complexity.

Beyond LOS, considerable variability existed in the perioperative outcomes reported across studies, including pain and analgesic use, complications, ambulation, return to diet, catheter and drain removal, readmission, and discharge disposition. This heterogeneity limits direct comparison between ERAS pathways and makes it difficult to determine which pathway components most consistently improve perioperative recovery. As the field matures, patient-reported outcomes, longitudinal function, independence, and continuity of oncologic therapy may provide complementary measures to determine whether improvements in perioperative recovery persist beyond hospital discharge.

Recent and ongoing studies suggest that ERAS research in orthopaedic oncology is shifting from pathway description to implementation, compliance, and patient-centred recovery [26,27,28]. A more recent prospective observational study of 300 patients undergoing surgery for benign and malignant bone and soft tissue tumours assessed compliance with 20 ERAS components and found an overall compliance rate of 84.5% [26]. Higher compliance was associated with shortened median hospital stay and fewer complications, emphasizing that pathway adherence may be as important as pathway design. Ongoing prospective studies in soft tissue sarcoma are also beginning to incorporate outcomes such as opioid consumption, patient-reported pain, function, quality of life, gait assessment, wearable-derived mobility, and daily step counts [27,28]. As ERAS pathways in orthopaedic oncology mature, an important unanswered question is whether improvements in perioperative process measures translate into sustained benefits beyond hospital discharge. Longitudinal patient-reported outcomes, functional measures, and objective mobility assessment may provide complementary information regarding recovery trajectories, while oncology-specific measures such as time to resumption of systemic therapy or radiotherapy may help determine whether streamlined perioperative recovery facilitates continuity of cancer care.

This scoping review has limitations. Only MEDLINE and Embase were searched, and inclusion was limited to English-language studies. As with any scoping review, it is possible that some studies were missed, although we mitigated this by using a librarian-developed broad search and duplicate screening to reduce the risk of missed studies and selection bias. The studies included were heterogeneous in population, diagnosis, anatomic site, ERAS elements, comparator groups, and outcome reporting. However, within the goals of the scoping study, our approach aimed to highlight the current breadth and depth of literature in the field to identify critical gaps and guide further investigation.

## 5. Conclusions

ERAS pathways in orthopaedic oncology remain at an early stage of development, with current evidence concentrated primarily in spine oncology. Although available studies suggest favourable perioperative outcomes, important gaps remain in non-spine sarcoma and appendicular MBD, with considerable heterogeneity in pathway design, implementation, and outcome reporting. Future work should focus on prospective disease- and procedure-specific implementation of adaptable ERAS pathways incorporating core principles, standardized perioperative outcomes, and measures of pathway adherence. Longitudinal evaluation may further determine whether improvements in perioperative recovery translate into sustained functional and oncologic benefits beyond hospital discharge.

## Figures and Tables

**Table 2:** Highlighted research gaps in the scoping review, with associated future research opportunities.

Research Gap	Future Research Priority
Evidence is predominantly retrospective, with few prospective comparative studies. Evidence is concentrated in spine and spinopelvic oncology, with limited appendicular sarcoma and no appendicular-only MBD studies. Considerable variability exists in the components used to define ERAS pathways. Pathway adherence and component compliance are inconsistently evaluated. LOS is the only consistent reported outcome and is influenced by patient, disease, and surgical complexity. Perioperative outcomes are inconsistently defined and reported. The relationship between improved perioperative recovery and longer-term functional or oncologic outcomes remains poorly understood.	Conduct prospective implementation and comparative studies of ERAS pathways in orthopaedic oncology. Develop and evaluate disease- and surgery-specific ERAS pathways for extremity/pelvic sarcoma and appendicular MBD. Establish core orthopaedic oncology ERAS principles while allowing adaptation according to diagnosis, treatment urgency, surgical complexity, prognosis, and functional goals. Prospectively measure adherence to individual ERAS elements and its relationship with perioperative recovery. Stratify LOS according to relevant clinical and procedural factors and report alongside other standardized perioperative outcomes. Develop a standardized core outcome set, including complications, pain and analgesic use, mobilization, readmission, and discharge outcomes. Determine whether improved perioperative recovery translates into sustained functional recovery, quality of life, independence, and continuity of oncologic therapy.

## Data Availability

The original contributions presented in this study are included in the article. Further inquiries can be directed to the corresponding author.

## Appendix A

### Appendix A.1. Search Strategy

Ovid MEDLINE(R) ALL <1946 to 9 August 2024>

1. exp Bone Neoplasms/ 138,981
2. (bone* adj2 (neoplas* or tumo?r*)).tw,kf. 23,428
3. exp Neoplasm Metastasis/ 225,457
4. ((neoplas* or tumo?r*) adj2 metas*).tw,kf. 80,931
5. ((tumo?r or neoplas*) adj2 metas*).tw,kf. 55,976
6. exp Sarcoma/ 154,046
7. exp Mast-Cell Sarcoma/ 2088
8. exp Sarcoma, Yoshida/ 1748
9. exp Sarcoma, Synovial/ 3567
10. exp Sarcoma, Clear Cell/ 693
11. exp Sarcoma 37/ 337
12. exp Histiocytic Sarcoma/ 995
13. exp Sarcoma 180/ 3095
14. exp Dendritic Cell Sarcoma, Interdigitating/ 87
15. exp Sarcoma, Small Cell/ 249
16. exp Sarcoma, Endometrial Stromal/ 850
17. exp Dendritic Cell Sarcoma, Follicular/ 319
18. exp Rous sarcoma virus/ 427
19. exp Sarcoma, Myeloid/ 1377 (granulitic sarcoma)
20. exp Sarcoma, Alveolar Soft Part/ 486
21. exp Sarcoma, Ewing/ 7973
22. exp Langerhans Cell Sarcoma/ 86
23. exp Sarcoma, Kaposi/ 11,046
24. sarcoma.tw,kf. 99,183
25. exp Multiple Myeloma/ 49,588
26. multiple myeloma.tw,kf. 48,964
27. 1 or 2 or 3 or 4 or 5 or 6 or 7 or 8 or 9 or 10 or 11 or 12 or 13 or 14 or 15 or 16 or 17 or 18 or 19 or 20 or 21 or 22 or 23 or 24 or 25 or 26,627,336
28. exp Enhanced Recovery After Surgery/ 1782
29. enhanced recovery after surgery.tw,kf. 4782
30. ERAS.tw,kf. 7681
31. early recovery pathway*.tw,kf. 6
32. early recover*.tw,kf. 4107
33. exp “Length of Stay”/ 105,989
34. (length adj2 stay).tw,kf. 87,602
35. (tim* adj2 discharg*).tw,kf. 9708
36. (expedited adj2 discharg*).tw,kf. 74
37. 28 or 29 or 30 or 31 or 32 or 33 or 34 or 35 or 36,176,778
38. exp Practice Guideline/ 31,962
39. guid*.tw,kf. 1,181,174
40. protocol*.tw,kf. 629,771
41. framework*.tw,kf. 456,065
42. pathway*.tw,kf. 1,554,923
43. 38 or 39 or 40 or 41 or 42 3,623,769
44. 27 and 37 and 43 170

### Appendix A.2. ERAS Components of Included Studies

**Table A1:** Summary of the ERAS components of included studies.

Author (Year)	Zhao (2021) [12]	Lyu (2020) [14]	Chakravarthy (2022) [1]	Michot (2015) [16]	Wang (2023) [5]	Yuh (2024) [11]	Lei (2023) [13]	Ma (2024) [15]	Nguyen (2021) [17]
Title	Effectiveness of enhanced recovery after surgery in the perioperative management of patients with bone surgery in China	Enhanced recovery after surgery pathway in patients with soft tissue sarcoma	Patient outcomes following implementation of an enhanced recovery after surgery pathway for patients with metastatic spine tumors	The introduction of early patient rehabilitation in surgery of soft tissue sarcoma and its impact on post-operative outcome	Enhanced recovery after surgery (ERAS) in sacral tumour surgery and comprehensive description of a multidisciplinary program: a prospective study in a specialized hospital in China	The iterative implementation of a comprehensive enhanced recovery after surgery protocol in all spinal surgery in Korea: a comparative analysis of clinical outcomes and medical costs between primary spinal tumors and degenerative spinal diseases	Treatment of patients with metastatic epidural spinal cord compression using an enhanced recovery after surgery program	Promoting postoperative recovery in patients with metastatic epidural spinal cord compression based on the concept of ERAS: a multicenter analysis of 304 patients	A multidisciplinary approach to complex oncological spine coverage in high-risk patients
ERAS Protocol	Enhanced recovery after major bone tumour surgery	Enhanced recovery after surgery protocol adapted for patients with soft tissue sarcoma at Brigham and Women’s Hospital	MSKCC Metastatic Spine Tumor ERAS Protocol	Adapted ERAS measures from recommendations of the ERAS^®^ pathway of the ERAS society to limb and trunk wall STS	ERAS Pathway for sacral tumour	The Seoul National University Hospital Spine Center Comprehensive ERAS Protocol	ERAS Program	Unique ERAS program created specifically for MESCC patients	Multidisciplinary protocol for complex oncological spine coverage in high-risk patients. Used perioperative optimization strategies.
Pre-admission					(1) Expectation setting (2) Individual condition preparation: Patient nutrition status, prohibition of smoking, and alcohol	(1) Patient education and counselling: w/written information material (2) Smoking and alcohol: Cessation 1 mo prior to surgery			
Preoperative	(1) Patient and family communication (2) Patient evaluation: Preoperative KPS, pain VAS score, anxiety and depression HADS score, nutritional status NRS 2002 score, VTE Caprini Risk Assessment Scale. (3) Nutritional intervention (4) Antithrombotic prophylaxis (5) Preventive analgesia (6) Blood management: HB raised to above 100 g/L (7) Liquid food 2 h before anesthesia and solid food 6 h before anesthesia for patients without aspiration risk	(1) Patient/family education (2) Medical optimization (3) Limiting fasting, made nil by mouth 6 h before operation (4) Carbohydrate-rich beverage (ClearFast^®^) given up to 2 h before operation (5) Regional block versus epidural placement	(1) Patient Education brochure given at last clinic visit prior to surgery: Expectations, Postoperative care (2) Day of Surgery: Gabapentin/pregabalin home dose, celecoxib 200 mg	(1) Patients given detailed information about the surgical program, and preventative measures (e.g., re-nutrition after weight loss) were taken (2) Sugary drinks were allowed up to two hours prior to anesthesia (3) Compressive stockings were put on, no thrombo-prophylactic medicines were given (4) No prophylactic antibiotics given (5) No urinary catheter set	(1) Patient education (2) Pulmonary function exercise (3) Digestive tract preparation (4) Foley’s catheterization (5) Nutrition loading (6) Fasting (7) Skin preparation (8) Allogeneic blood preparation	(1) Fasting protocol	(1) Comprehensive medical education (2) Preoperative assessment (3) Nutrition support (4) Prevention of venous thrombosis (5) Treatment of anaemia before operation (6) Measures of preventing bleeding (7) Preventative pain management (8) Diet management (9) Intestinal preparation (10) Respiratory function training	(1) Medical education (2) Preoperative education (3) Nutritional support (4) Prevention of venous thrombosis (5) Treatment of anaemia before surgery (6) Intestinal preparation (7) Pre-emptive analgesia (8) Training for respiratory function (9) Mental healthcare	(1) If pre-albumin < 16 or albumin < 3.5, give oral protein supplement. Consider tube feed if patient cannot tolerate PO intake. (2) If perioperative steroid is used, give vitamin A supplementation and micronutrients (zinc and selenium). (3) If patient had prior radiation exposure, give micronutrients (zinc and selenium). (4) If a patient has poor skin quality or one of the above risk factors, consider incisional negative pressure wound therapy (NPWT).
Intraoperative	(1) General anesthesia (2) Local incision anesthesia (3) Control bleeding (4) Pain management (5) Infusion restriction (6) ICU and extubation	(1) Opioid-sparing, multimodal analgesia (2) Euvolaemia (3) Normothermia (4) Normoglycaemia (5) Minimize tubes and drains	(1) Goal-Directed Fluid Therapy (2) Analgesics	(1) General anesthesia was complemented by administering a loco-regional analgesic for pain prevention (2) Avoided tourniquets to prevent ischemia (3) Performed gentle tissue-, nerve-, and vessel-sparing dissection (4) Used tension-free wound closure or if needed tissue flaps (5) Wound drainage was omitted whenever possible and in order to avoid dead spaces favouring fluid collections, a compressive bandaging (wrapping) was applied mainly to extremity sarcoma, whereas stitches securing the skin to underlying tissues were applied mainly to trunk wall sarcoma (6) No splint was set-up, allowing joints to move freely (7) No patient stayed in the intensive care unit (ICU)	(1) Prophylactic antibiotics (2) Multimodal analgesia (3) Surgical plan (4) Multidisciplinary cooperation (5) Wound drainage	(1) Standard anesthesia protocol (2) Antimicrobial prophylaxis (3) Surgical techniques (4) Drains (5) Urinary drainage	(1) Prevention of infection (2) Anesthesia method (3) Operation method (4) Drainage method (5) Body temperature monitoring and insulation (6) Pre-emptive analgesia (7) Local anesthesia of incision (8) Preventive hemostasis with tranexamic acid (9) Optimization of intraoperative fluid replacement (10) Prevention of postoperative nausea and vomiting	(1) Prevention of infection (2) Anesthesia (3) Surgery: Operation type (4) Incision local anesthesia (5) Monitoring and insulation of body temperature (6) Hemostasis and blood transfusion (7) Goal-directed fluid treatment (8) Surgery drainage (9) Prevention of nausea and vomiting	(1) If the spinal defect was not appropriate for flap coverage, the plastic surgery team was on stand-by for intraoperative consult if needed.
Postoperative	(1) Diet (2) Infusion restriction (3) Pain management (4) Blood management (5) Urinary catheter removal (6) PONV (7) Early mobilization	(1) Early nutrition (2) Early mobilization (out of bed) (3) Minimize intravenous fluids, hep-lock fluids at 12 h (4) Management of nausea and vomiting (5) Minimize opioids	(1) Discontinue urinary catheter (2) Regular diet (3) Ambulation, PT (4) Analgesia	(1) Loco-regional analgesics continued postoperatively and subsequently replaced by oral analgesics (2) Involving physiotherapy to help patients to reacquire their autonomy in the postoperative period. With reassurance and technical support patients began to mobilize as early as day 0 (3) Bandages removed after day 1 and dressing after day 2, leaving the suture open thereafter (4) Patients were allowed to have showers (5) Stockings were kept until the patients were discharged (6) Patients were discharged as soon as they were able to walk autonomously without pain	(1) Early semi-sitting position (2) Early oral intake (3) Multimodal analgesia (4) Prophylactic antibiotics (5) Prevention of nausea and vomiting (6) Early mobilization (7) Management of wound	(1) Local anesthetic techniques (2) PONV prevention (3) Multimodal opioid-sparing analgesia (4) Early mobilization (5) Early oral nutrition (6) Feedback survey from patients (7) Education before discharge	(1) Multimodal analgesia (2) Diet management (3) Early removal of urinary catheter (4) Drainage management (5) Prevention of venous thromboembolism (6) Management of postoperative malignant vomiting (7) Management of postoperative abdominal distension and constipation (8) Fluid management (9) Rehabilitation training (10) Management and treatment of delirium	(1) Pain management (2) Diet management (3) Urinary catheter management (4) Removal of the incision drainage tube (5) Preventive measures for venous thromboembolism (6) Treatment options for postoperative malignant vomiting (7) Postoperative abdominal distension and constipation (8) Early rehabilitation training (9) Delirium management and treatment	
Discharge	(1) Patient assessment: Preoperative KPS, pain VAS score, anxiety and depression HADS score (2) Other assessments: complications, LOS			(1) After discharge, patients were advised to continue with the apprised self-re-education technique. (2) Physiotherapy was not prescribed in order not to hamper wound healing during the first four postoperative weeks. (3) Oral analgesics, mainly paracetamol completed by tramadol if needed, were continued for few days with no other medical treatment.					
Follow-Up	(1) Patient evaluation 30 d after discharge			(1) A postoperative visit was organized four weeks after surgery. (2) In the event of a complication, especially seroma formation, patients were advised to contact the outpatient consultation unit for drainage.	(1) Effective follow-up: Telephonic and/or Wechat follow-up are performed at 48 h and 1 week after discharge.	(1) Postop 2 wks: Check the surgical wound and stitch out at a local medical centre (2) Postop 1 mo: Outpatient clinic visit; plan additional treatment if needed (3) Hotline: Direct calls to the Seoul National University Hospital Spine Center hotline			
Terms	BMI: body mass index; ERAS: Enhanced Recovery After Surgery; KPS: Karnofsky Performance Status; VAS: visual analog scale; HADS: Hospital Anxiety and Depression Scale					GW = general ward; IV = intravenous; POD = postoperative day; PONV = postoperative nausea and vomiting; prep = preparation; NSAID = nonsteroidal anti-inflammatory drug; prn = pro re nata; TID = three times a day; TIVA = total intravenous analgesia.		Abbreviations: ERAS, Enhanced Recovery After Surgery; VAS, visual analogue scale; PCA, patient-controlled analgesia; ICP, intermittent pneumatic compression; PONV, postoperative nausea and vomiting	
Notes				No concrete ERAS protocol provided, details were summarized from methods section					Not an ERAS protocol
